# Methylation and algorithms in biological aging: a scoping review

**DOI:** 10.3389/fragi.2025.1682873

**Published:** 2025-12-18

**Authors:** Alison Ziesel, Jennifer Reeves, Anastasia Mallidou, Lorelei Newton, Ryan E. Rhodes, Jie Zhang, Theone Paterson, Hosna Jabbari

**Affiliations:** 1 Department of Biomedical Engineering, University of Alberta, Edmonton, AB, Canada; 2 Department of Psychology, University of Victoria, Victoria, BC, Canada; 3 School of Nursing, University of Victoria, Victoria, BC, Canada; 4 Institute on Aging and Lifelong Health, University of Victoria, Victoria, BC, Canada; 5 School of Exercise Science, Physical and Health Education, University of Victoria, Victoria, BC, Canada; 6 Gustavson School of Business, University of Victoria, Victoria, BC, Canada

**Keywords:** biological age, biological clocks, epigenomics, methylomics, biomarkers

## Abstract

The role of DNA methylation in the process of biological aging is a particularly active area of research, where methylation changes may be a consequence or a driver in the deviation between biological and chronological age. We employ a scoping review strategy to analyze the results of 435 relevant research papers, 167 of which employed methylation-based strategies to interrogate biological age. Our work details the progression and refinement of these strategies over time, as well as the development of novel methylation-based clocks and algorithmic methods. Our chosen review strategy allows for the identification of research findings consistent and discordant with one another, as well as focusing on exciting, potential research areas regarding measurement, calculation, and assessment of epigenetic biological age.

## 1 Introduction

Organisms age in a process related to but not strictly tied to their chronological age. This difference in aging rate is referred to as biological age, and it influences development, maturation and later life. As there is a difference between chronological age and the aging rate, age-related disease risk and developmental time points may not be correctly assessed by chronological age exclusively. Biological aging may reflect an acceleration or deceleration in the aging process, and the difference between biological and chronological age is likely to diverge with time. Correct assessment of the biological aging process is critical to accurate determination of both development and disease progression.

Changes that underlie and indicate biological aging occur at every scope, from subcellular to organismal levels; diverse features have been positively correlated with the rate of aging, and so it is possible to assess the biological aging process cellularly, individually, or at the population level. When biological aging is accelerated, individuals may be at a greater premature risk of age-related disease. The capability to analyze populations and individuals for biological age changes could make possible both surveillance strategies to detect pre-disease states and early treatment to ameliorate those states before they become untreatable. Timely, precise biological age assessment is essential to identifying early disease susceptibility and time points where treatment is maximally effective for the promotion of continuing good health in individuals and populations.

The questions of aging and aging rates have preoccupied scientists for centuries, with organized studies beginning in the mid-twentieth century. Studies conducted in the 1950s describing a “functional age” parameter were performed by McFarland (McFarland, 1958) and were later revisited by Dirken and Furukawa (Dirken, 1972; Furukawa et al., 1975), while some thinkers questioned biological age and its utility in characterizing development (Costa and McCrae, 1980; Dean and Morgan, 1988). Early studies frequently employed physical and clinical parameters, and these features persist in the study of biological aging to this day. Molecular and genetic features including telomere length attrition (Levy et al., 1992) and mitochondrial DNA (mtDNA) copy number decline (Welle et al., 2003; Cree et al., 2008) were observed in the early 1990s and 2000s. Initially reported as early as 1967, both global and site-specific age-related methylation changes were revisited in the early twenty first century, leading to a greater focus on these epigenetic changes in subsequent studies (Berdyshev et al., 1967; Romanov and Vanyushin, 1981; Fernandez et al., 2012; Bocklandt et al., 2011). This led to the development of the first epigenetic clocks in the 2010s, which undertake to predict age solely from the methylation status of a subset of cytosine-phosphate-guanosine (CpG) genomic sites (Hannum et al., 2013; Horvath, 2013). Second and third generation epigenetic clocks followed, many of which incorporate additional, non-methylation data to predict biological age and in some cases, mortality (Belsky et al., 2015; Levine et al., 2018; Lu et al., 2019; Belsky et al., 2020).

The previous paragraph represents a high level overview of the discoveries and novel strategic approaches developed for the characterization of biological age. While reviews exist that examine subsets of work done during this period, we have not identified any writing that produced a comprehensive summary of the work analyzing molecular biological aging features along with those clocks and algorithms developed to interpret those data. We have prepared two reviews: this work focusing on the epigenetic features and algorithms, and a second review considering all non-methylation-based, molecular approaches biological age calculation. Our work addresses a critical gap in the literature by unifying a fragmented field – one where most studies focus narrowly on specific markers or algorithms – into a comprehensive synthesis of the molecular features of biological aging and related algorithms. By examining 435 studies over the 12 year period and using established frameworks (Arksey and O’Malley, 2005; Tricco et al., 2018), our pair of reviews systematically integrates molecular, epigenetic, and algorithmic approaches. Unlike previous reviews, these provide a broad, methodologically transparent review of biological aging measures, and also lay the groundwork for developing a more systematic, focused review of socioeconomic, clinical, and demographic factors in biological aging. In doing so, we overcome the challenges that have deterred other scholars – such as the field’s rapid expansion, data fragmentation, interdisciplinary complexity, and methodological variability – and contribute a credible, impactful map of current knowledge, key limitations, and future directions for research.

This scoping review addresses questions regarding epigenetic measurement of the differential between chronological and biological age that have recently arisen. We have chosen a scoping review strategy for its strengths in identifying available evidence, mapping domain knowledge and uncovering gaps in available evidence. Scoping reviews are a strong choice to prepare for a more focused systematic review, and are not intended as a meta-analysis or critical assessment of the studies reviewed. We aim to identify those outstanding critical questions within the larger field of epigenetic indicators of biological aging, with additional focus on algorithmic approaches in biological age calculation. This scoping review spans a large body of research, and as such the reader may wish to pursue specific subsections of this work that are particularly relevant to their interests.

## 2 Materials and methods

This review adhered to guidelines in the PRISMA Extension for Scoping Reviews (Tricco et al., 2018), and was guided by Arksey and O’Malley’s methodological framework (Arksey and O’Malley, 2005).

### 2.1 Search strategy

Four databases were searched from January 2011 to June 2023: PsycINFO, CINAHL, PubMed, and SPORTDiscus. Search terms included terms associated with biological and chronological aging, with all terms searched in the abstract and title. In addition, searches included relevant MeSH headings. Limits were applied to our search findings, specifically publication date (January 2011 to end of June 2023), subject (humans), age (adults aged 18 or older), and publication language (English). Refer to Supplementary Appendix A for the full search strategy and search terms used.

### 2.2 Eligibility criteria

#### 2.2.1 Population

To be eligible for inclusion, studies considered only human participants, aged 18 or older. These studies were included if they examined healthy adults or adults with specific health conditions (e.g., cancer). Studies including use of human-derived cell lines and human remains were retained.

#### 2.2.2 Outcomes

Studies chosen for inclusion measured chronological age and compare either a calculated biological age or a molecular indicator of biological aging (e.g., telomere length) with chronological age. Studies which examined the biological aging of only a specific organ were excluded. Only those biological age measures based on methylation-derived data were included in this work.

#### 2.2.3 Design

Empirical primary research studies were considered for inclusion if they were peer-reviewed and published in a journal. Non-research, theses, dissertations, theoretical papers, seminar papers, case studies, book chapters, study protocols, review articles, and articles in-press during the period assessed were excluded. By emphasizing published, peer-reviewed studies, this scoping review prioritizes data quality and transparency. This deliberate choice ensures that the synthesis reflects well-vetted research and established patterns. By emphasizing methodological rigor, reliability, and credibility, this review provides a solid foundation for future inquiries and expansion of the field’s knowledge base. We did not impose any limits regarding study size or number of participants in a given study; even small or modestly sized studies may reveal important insights for this field.

#### 2.2.4 Study selection and data charting

Reviewers used the software platform Covidence (2024) to screen articles based on title and abstract. Two reviewers examined each title and abstract, and conflicts were resolved by a third reviewer. Full text articles were then screened according to described inclusion criteria. Conflicts were resolved by the three reviewers re-examining and discussing the article in question in the context of our inclusion criteria.

Data was extracted by reviewers, including the aims, population, methodology, and findings of the studies. Extracted data were then verified by one additional reviewer.

#### 2.2.5 Data synthesis

Following data extraction, a composite list of all outcomes compared to chronological and/or biological indicators of aging was composed. Categories of those indicators employed were created resulting in 10 groups, which are described in Table 1. Due to the breadth of information summarized by our work, we have focused on the underlying methylation-based features of biological aging, and the extant algorithms and clocks established for determining biological age.

**TABLE 1:** Categories of biological aging data analyzed in this review.

Categories
Non-Clock/Whole genome methylation
Horvath’s pan-tissue clock
Horvath’s Skin and Blood clock
Hannum’s clock
PhenoAge
GrimAge
Weidner’s clock
DunedinPoAm
Novel and specific clocks

## 3 Results

### 3.1 Study characteristics

**FIGURE 1:**
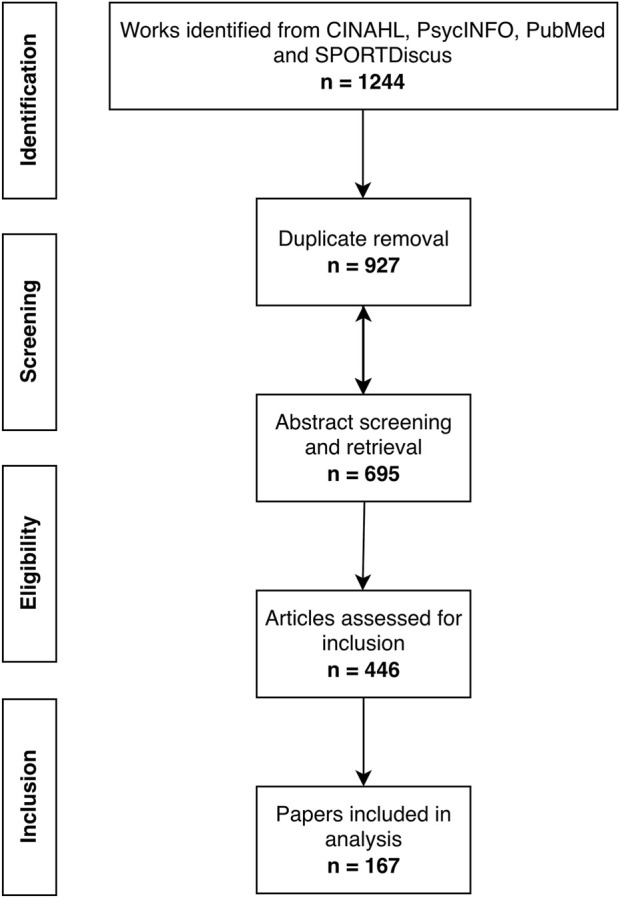
Flowchart detailing paper selection and filtration.

We identified 1,244 works from four research databases during our data collection phase, from which 317 duplicates were removed (Figure 1). The remaining 927 records were screened for title and abstract, and 232 were removed. We sought 695 full text articles, of which one could not be retrieved, and excluded another 248 based on our inclusion and exclusion criteria, resulting in 446 articles for our analyses. We excluded a further eleven papers as using exclusively non-molecular or non-biological metrics of biological aging, resulting in 435 papers; of these, 167 included methylation-based biomarkers of aging, which were analyzed in this review.

The total number of participants in the included manuscripts is 592,816; due to overlap in datasets between some studies, the number of unique participants is lower. A total of 90 studies used data from a non-unique dataset, the most frequently used datasets included the Health and Retirement Study (HRS) in 10, the National Health and Nutrition Examination Survey (NHANES) in two manuscripts, the Dunedin Cohort in two, the Normative Aging Study (NAS) population in six, and the Lothian Birth Cohorts of 1921 and/or 1936 in seven. Twenty three studies used data from multiple datasets. Remaining studies used populations unique to those publications.

The included studies focused on different population parameters. Some studies selected participants based on age, with many studies (64 total) considering middle-aged and older adults. Seven studies focused on young adults, 64 studies included young and middle-aged adults, 15 studies focused solely on middle-aged adults, and eleven studies included long-lived individuals including centenarians. Studies often focused on health outcomes, with eight studies including individuals with mental health and cognitive conditions, 19 studies focusing on specific physical health conditions including but not limited to chronic cardiovascular disease, ischemic stroke, or infertility. Seven studies included cancer patients and survivors, and three studies examined HIV positive individuals. Twelve studies examined specific lifestyle parameters, including one which focused on athletes, one on tobacco smokers, and one on long term meditators. Career influence on biological age was examined in five studies, while seven studies examined differential aging rates in twins. Some studies specifically recruited by sex, including 26 studies involving females only, and 10 studies with only males. A plurality of the included studies examined the general population, rather than focusing on parameter-specific subpopulations. Table 2 summarizes the study characteristics of those works reviewed here.

**TABLE 2:** Characteristics of those studies analyzed in this review. NHANES: National Health and Nutrition Examination Survey; HRS Health and Retirement Study; NAS Normative Aging Study; LBC21/36 Lothian Birth Cohort(s) of 1921 and/or 1936.

Characteristic	# Of studies	Percentage
Location		
United States	82	49.1%
United Kingdom	13	16.2%
China	5	3.0%
Other countries	65	38.9%
Multiple countries (including above)	13	7.8%
Not reported	2	1.2%
Age		
All adults (18–99)	30	18.0%
Young adults (18–29)	53	31.7%
Adults (30–59)	124	74.3%
Older adults (60–99)	127	76.0%
Centenarians (100 +)	11	6.6%
Gender		
Mixed	131	78.4%
Female only	26	15.6%
Male only	10	6.0%
Study populations		
NHANES	2	1.2%
HRS	10	6.0%
Dunedin cohort	2	1.2%
NAS	6	3.6%
LBC21/36	7	4.2%
Other non-unique	90	53.9%
Multiple datasets	23	13.8%
Unique datasets	55	32.9%
Outcomes		
Mental health outcomes	8	4.8%
Physical health outcomes	22	13.2%
No outcome focus	137	82.0%
Lifestyle parameters		
Athletes	1	0.6%
Tobacco smokers	1	0.6%
Career choice	5	3.0%
Other lifestyle parameters	10	6.0%
No specific parameters	123	73.7%

**FIGURE 2:**
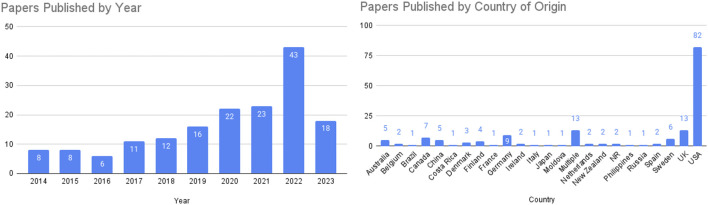
Left panel: papers published per year analyzed in this review, with 2023 data collection ending in June. Right panel: country of origin for studies included in this review, which provides information regarding the ethnic and racial composition of study populations included in this work.

Publication of works on biological aging increased steadily during the period of time reviewed (Figure 2 left panel). Studies included were conducted in 22 countries, with the most frequent being the United States (82 manuscripts), followed by 13 in the United Kingdom, and 5 in China. Thirteen studies occurred in multiple countries, and two studies did not report the country they occurred in. Figure 2 (right panel) details the breakdown by country for those works included in this manuscript.

**FIGURE 3:**
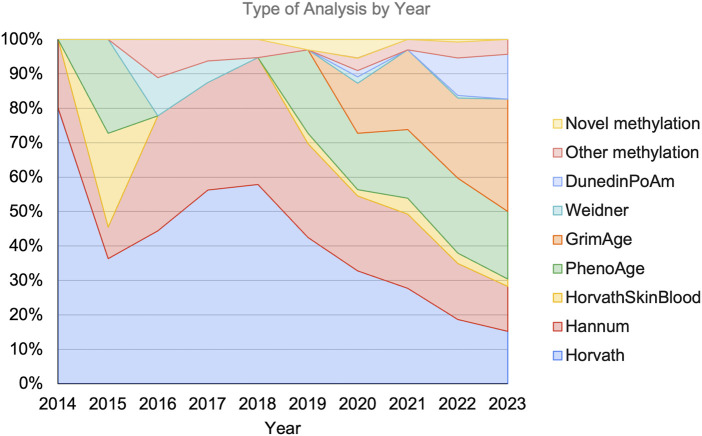
Types of biological aging analyses conducted per year as a percentage of works published in that year.

All manuscripts reviewed included biological age assessment, sometimes employing multiple metrics, with CpG methylation-based epigenetic clocks used 226 times in these studies, although some publications made use of multiple CpG-based clocks. Some published studies describe clocks developed based on novel CpG collections. Figure 3 describes the breakdown of analyses employed in papers published per year.

### 3.2 Molecular biology features

#### 3.2.1 Non-clock and genome-wide methylation

##### 3.2.1.1 Non-clock CpG sites

In addition to methylated CpGs that are employed by various epigenetic clocks, CpG methylation globally and at specific loci is also studied in biological aging. In 2012, the first paper identifying age-associated CpG methylation within the gene ELOVL2 was published, finding an increase in methylation with increasing age (Garagnani et al., 2012). A study of rRNA locus methylation found one CpG to significantly decrease in methylation with chronological age, while an additional CpG site was associated with impaired cognitive performance and a higher mortality risk (D’Aquila et al., 2017). The authors followed up with a more expansive study in 2019, finding hypermethylation of a CpG contained within the RAB32 gene, and hypomethylation of a CpG in the RHOT2 gene, were associated with increasing chronological age (D’Aquila et al., 2019). More recently, in a set of post-COVID19 infection and COVID19-free individuals, biological age as determined by a set of four CpGs contained within the genes ASPA, EDARADD, ELOVL2 and PDE4C found that the post-COVID19 infection participants had a biological age 9 years older than uninfected counterparts, with the effect being more significant in younger individuals (Bekaert et al., 2015; Mongelli et al., 2021). Kim and colleagues conducted a twin study, looking for CpGs associated with increased frailty in the elderly, and identified a CpG contained within the gene PCDHGA3, as well as several regions containing multiple CpGs (Kim et al., 2018). A 2015 paper describing the pattern of CpG-reported tobacco and alcohol consumption in conjunction with biological age as determined by Hannum’s clock found that increased cigarette smoking leads to an asymptotic increase in biological aging acceleration, while alcohol consumption showed increased biological aging at consumption extremes, with modest age deceleration at moderate consumption levels (Beach et al., 2015). In a study of the effects of a relaxation program on biological age between 10 healthy and 20 myocardial infarction survivor participants, methylation of five genes were measured and showed that for only the healthy participants these chronological age-correlated genes’ methylation state reverted to a younger state (Pavanello et al., 2019). Ross and colleagues observed the association of methylation of PAI-1 associated with chronological age, and that biological age measured by that metric decreases over the course of pregnancy; they further found that pregnancy-related increases in body mass index (BMI) led to post-birth decreases in biological age, but that no decrease in BMI in the post-birth year was associated with higher biological age in a study of 35 women (Ross et al., 2020).

Using Illumina Infinium MethylationEPIC chips in an epigenome-wide association study (EWAS), Tajuddin et al. report an unexpectedly wide variety of CpGs differentially methylated in African Americans relative to white subjects (Tajuddin et al., 2019). 4,930 CpGs were found to be differentially methylated in African Americans compared to 469 found in European ancestry white participants of the Healthy Aging in Neighborhoods of Diversity across the Life Span (HANDLS) study. Only 301 of those CpGs were in common between the two racial groups, and this study identified novel differentially methylated CpGs in both racial groups. A study of methylation at CpG sites in the genes ASPA, ITGA2B and PDE4C found that following 12 months of vitamin B supplementation, ASPA CpG methylation, which typically declines with age, was significantly higher than in control subjects, while PDE4C, which normally increases with age, trended even higher following vitamin supplementation in 63 participants (Obeid et al., 2018). In a study on the Lothian Birth Cohort of 1921, Marioni and colleagues identified a CpG highly correlated with facial aging, along with an additional 32 CpGs correlated with facial age and increased mortality risk (Marioni et al., 2018).

##### 3.2.1.2 Genome-wide DNA methylation

DNA methylation at sites not associated with established epigenetic clocks and global levels of genomic DNA methylation may be associated with biological aging. A study of genome-wide average methylation in a group of 479 healthy women found no association with chronological age or other methylation-based clock measures of biological age, but did find a negative association with the number of live births, and a positive association with age at first live birth (Chen et al., 2019). A cross-sectional cohort focused on the most and least socioeconomically deprived individuals in the Psychological, Social, and Biological Determinants of Ill Health (pSoBid) study revealed that global DNA methylation was significantly negatively correlated with inorganic phosphate levels, which is associated with poorer diet, lower socioeconomic status and increased cellular inflammatory status (McClelland et al., 2016). In a more specific study of nine genes, it was determined that seven of those genes showed aging-related patterns of methylation: methylation of the genes GCR, iNOS and TLR2 decreased, while methylation of IFN γ , F3, CRAT and OGG increased with increasing chronological age in a study of NAS participants (Madrigano et al., 2012).

### 3.3 Biological aging algorithms and clocks

#### 3.3.1 Horvath’s pan-tissue clock

Perhaps the best known epigenetic clock is the Horvath pan-tissue clock, developed in 2013 using 51 healthy cell types and tissues, and elastic net regression to predict biological age (Horvath, 2013). The Horvath pan-tissue clock calculates biological age based on the methylation status of 353 CpGs and exhibits high correlation with chronological age and substantial performance across different biopsied tissues and cells. The Horvath pan-tissue clock is a first generation epigenetic clock, based exclusively on DNA methylation and represents a major step forward in the biological age measurement. In the 108 papers published in the review window using the Horvath pan-tissue clock, for those that reported correlation with chronological age all reported a substantial positive correlation. Further, 49 or 44.5% of those papers reported use of the Houseman algorithm for cell type heterogeneity correction to improve biological age estimation accuracy (Houseman et al., 2012). This clock also exhibits moderate to high correlation with the Hannum epigenetic clock, the Horvath skin and blood clock, the Weidner clock, and with the second and third generation clocks, PhenoAge, GrimAge and Dunedin Pace of Aging (Belsky et al., 2018a; Belsky et al., 2020; Chen M. et al., 2019; Dada et al., 2021; Dugué et al., 2018; Esteban-Cantos et al., 2021; Li et al., 2020; McCrory et al., 2020; Nwanaji-Enwerem et al., 2020; Nwanaji-Enwerem et al., 2021a; Roetker et al., 2018; Roshandel et al., 2020; Soriano-Tárraga et al., 2016). This clock also correlates negatively with mtDNA copy number (Dolcini et al., 2020). However, the Horvath pan-tissue clock does correlate with measures of facial aging (Belsky et al., 2018b; Marioni et al., 2018). There may be a gender-specific component, as some reports include findings of faster aging observed in men than in women (Bao et al., 2022; Baranyi et al., 2022; Beydoun et al., 2020; Beydoun et al., 2022; Dugué et al., 2018; Faul et al., 2023; Kankaanpää et al., 2022b; Kuzawa et al., 2022; Lind L. et al., 2018; McCrory et al., 2020; O’Shea et al., 2022; Sugden et al., 2022). One report detailed the reverse finding (Martin et al., 2021). Ethnicity may also play a role: epigenetic aging by Horvath’s clock was found to be accelerated in white versus African American study participants, and versus Hispanic study participants, but these findings were not universally reported (Beydoun et al., 2022; Faul et al., 2023; Graf et al., 2022; Tajuddin et al., 2019). The rate of aging by this clock may be partially heritable, as a study of twins and non-twin sister pairs found that monozygotic twins had greater correlation of the differential between chronological age and biological age than did dizygotic twins or non-twin sibling pairs, and a study of 4,658 elderly adults estimated the heritability of that differential at 43% (Li et al., 2015; Marioni et al., 2015).

The Horvath pan-tissue clock has also been tested on cell lines, and an experiment involving long-term cultured fibroblasts found that epigenetic age accumulated as the cells grew but ceased to increase once cells reached senescence (Sturm et al., 2019). While the Horvath pan-tissue clock was developed while the Illumina Infinium Human Methylation 27 k and 450 k BeadChips were predominant, a newer chip, the Infinium MethylationEPIC BeadChip later became available, boasting an increased 850,000 CpG sites interrogated. A 2018 study found that, despite the absence of 19 of the 353 CpGs of the Horvath pan-tissue clock, MethylationEPIC BeadChips did not suffer from decreased performance in epigenetic age prediction (McEwen et al., 2018). Studies of the accuracy of the Horvath pan-tissue clock has found a tendency to underestimate middle-aged and older individuals: a study of middle-aged twins found a systematic underestimation of age relative to chronological age, and cortex samples biopsied from individuals greater than 60 years of age were similarly underestimated (Shireby et al., 2020; Starnawska et al., 2017).

Biological age as measured by the Horvath pan-tissue clock has been repeatedly associated with mortality risk. In a study of 378 twins, it was found that among the oldest twins studied, the twin with the oldest biological age by Horvath’s clock was the first twin to die in 69% of cases, which corresponded to a more than three times the risk of mortality as compared to their twin (Christiansen et al., 2016). In a study including the Lothian Cohorts of 1921 and 1936, a 5 year increase in age acceleration corresponded to an increase in mortality risk of 9% (Marioni et al., 2015). A 2020 study found that a one standard deviation increase in biological age resulted in a 17% increase to mortality risk, a finding that was further elevated in ever smokers (Li et al., 2020). This correspondence with mortality risk was not always observed: Gao and colleagues developed a mortality risk score that did not associate with Horvath clock age acceleration, while a study involving participants from the Louisiana Healthy Aging Study cohort found that frailty index was a better predictor of mortality than both chronological age and Horvath biological age, and a further study found no sensitivity to age-related decline in clinical parameters (Gao et al., 2019; Kim et al., 2017; McCrory et al., 2021). An additional study considering the Lothian Birth Cohort of 1936 found that Horvath’s biological age in conjunction with neuroimaging-based age difference was a better predictor of mortality than either individual measure (Cole et al., 2018). Morbidity is also predicted by Horvath’s biological age: accelerated aging was associated with multimorbidity, but other studies found no association with physical frailty (Crimmins, 2020; Cruz-Almeida et al., 2019; Gale et al., 2018b; Gale et al., 2018a). However, a 2022 study of postmenopausal women found participants less likely to achieve age 90 with intact mobility as their Horvath’s age acceleration increased (Jain et al., 2022). In a study of young adults and nonagenarians, cytomegalovirus (CMV) seropositivity was associated with elevated Horvath age in both groups (Kananen et al., 2015).

Beyond mortality and morbidity, Horvath pan-tissue age acceleration associates with chronic disease conditions. In amyotrophic lateral sclerosis (ALS) patients, researchers found a highly significant association between age acceleration and age at ALS onset, and greater age acceleration was associated with decreased survival (Zhang et al., 2020). HIV patients show an increased Horvath age acceleration that is reversed following antiretroviral therapy (Esteban-Cantos et al., 2021; Schantell et al., 2022). Female multiple sclerosis patients, compared to healthy females and all males, show a negative age acceleration by Horvath’s clock, although the authors also observe a change in blood cell composition in these patients that may be a contributory factor (Theodoropoulou et al., 2019). Among study participants with and without a mutation in the LRRK2 gene, Parkinson’s patients were found to be biologically older by Horvath’s clock, and was further associated with an earlier age of onset, but not greater disease severity (Tang et al., 2022). Non-alcoholic steatohepatitis patients with phase 3 fibrosis exhibit a higher epigenetic age, and age acceleration was significantly correlated with hepatic collagen content in a study of 44 patients (Loomba et al., 2018). Roetker and colleagues report that increased carotid intima-medial thickness positively associated with Horvath age acceleration, while HDL cholesterol levels associated negatively (Roetker et al., 2018). They also observe increased hazards ratios for cardiovascular disease and related mortality in 10 years with increased age acceleration. Age acceleration was significantly related to cardiovascular disease, with an increased risk of 4% of a cardiovascular disease event occurring with each year of increased biological age (Lind et al., 2018). Individuals with increased pulse or systolic blood pressure, and women with hypertension were reported as having elevated epigenetic age in a 2022 study (Xiao et al., 2022). Conversely, a relatively large study involving the Rhineland Study population found no association with cardiovascular disease, and a separate study found no association with Horvath’s biological age was observed in ischemic stroke patients (Fox et al., 2023; Soriano-Tárraga et al., 2016). A study of the Melbourne Collaborative Cohort Study population found an association with increased age acceleration and diabetes, but a much smaller study group found no such association (Dugué et al., 2018; Vyas et al., 2019).

Physical parameters have been shown to associate with Horvath pan-tissue age acceleration, in particular BMI and related measures (Chen et al., 2019; Faul et al., 2023; Fiorito et al., 2019; Grant et al., 2017; Kresovich et al., 2021b; Li et al., 2019; Ross et al., 2020; Sillanpää et al., 2018). However, a twin study published in 2022, confirming that women were biologically younger than men according to Horvath’s clock, observed that that phenomenon was partially mediated through BMI, with men in the study population typically having higher BMI than women (Kankaanpää et al., 2022b). In women among the SAPALDIA and ECHRS cohorts, FEV1 and forced vital capacity (FVC) were associated with age acceleration, while in men there was an association with FVC in the follow-up time points only (Rezwan et al., 2020). Levels of physical activity associate negatively with age acceleration in some, but not all, studies (Fiorito et al., 2019; Roshandel et al., 2020; Spartano et al., 2023; Vetter et al., 2022a; Vyas et al., 2019). Among veterans diagnosed with COPD, it was observed that biological age was inversely associated with performance in the 6 minute walk distance test (Wan et al., 2021).

In a study involving bariatric surgery patients, reduced biological age was observed in patients following surgery (Fraszczyk et al., 2020). In a study of overweight and obese breast cancer survivors weight loss, metformin treatment, or combination therapy did not result in an improvement in Horvath’s biological age, however (Nwanaji-Enwerem et al., 2021a). Dietary improvement also resulted in biological age deceleration: a randomized clinical dietary trial resulted in decreased biological age after 8 weeks of intervention in 43 men, and a Mediterranean-style diet trial led to improvements in biological age among Polish participants, particularly women, after 1 year (Fitzgerald et al., 2021; Gensous et al., 2020). However, other studies of the Mediterranean diet, the DASH diet, and the CALERIE trial found no significant changes in biological age (Kresovich et al., 2022; Waziry et al., 2023). A study involving the Melbourne Collaborative Cohort found elevated meat consumption was associated with increased Horvath age acceleration (Dugué et al., 2018). Additionally, dietary supplementation has been found to influence biological age. A 2019 randomized clinical trial of 51 participants and a larger 2022 study both found vitamin D supplementation resulted in a decreased age acceleration among participants (Chen L. et al., 2019; Vetter et al., 2022b). Personal environment is relevant to biological age, as observed in a study of the Detroit Neighborhood Health Study: neighborhood quality was found to be associated with accelerated aging (Martin et al., 2021). Two studies found a negative association between educational attainment and epigenetic age acceleration (Dugué et al., 2018; Fiorito et al., 2019). Other work reported no association with educational attainment, childhood and adulthood socioeconomic status and changes in class, and household earnings (Bao et al., 2022; George et al., 2021; Schmitz et al., 2022). One study identified an improvement in epigenetic age associated with having endured childhood financial hardship (Faul et al., 2023). A 2022 report identified a deceleration of biological age among married compared to never married and divorced study participants (Bao et al., 2022). No impact from spousal, child, friend, other family support or contact frequency was observed on Horvath biological age in a study examining the HRS population (Hillmann et al., 2023).

Alcohol consumption and substance abuse disorder diagnosis do not exhibit a correlation with Horvath’s biological age (Bøstrand et al., 2022; Cabrera-Mendoza et al., 2023; Kresovich et al., 2021a). In several studies current or past smoking associates with increased biological age by the pan-tissue clock (Faul et al., 2023; Lei et al., 2017; Vyas et al., 2019). Similarly, airborne pollution is associated with increasing biological age acceleration: elevated ${\text{PM}}_{2.5}$ , sulfate and ammonium exposure were associated with increased age acceleration (Nwanaji-Enwerem et al., 2017). The finding of ${\text{PM}}_{2.5}$ exposure leading to increased age acceleration was also found in a 2016 study that combined the NAS and KORA F4 study cohorts; this study also found that in men, increased ${\text{PM}}_{10}$ exposure, while in women, black carbon and nitric oxides exposure were associated with increased age acceleration (Ward-Caviness et al., 2016). In a 2022 study of 26 participants, increases in immediately recent ${\text{PM}}_{2.5}$ exposure prior to blood draw resulted in biological age increase (Gao et al., 2022a). Among the Lothian Birth Cohort of 1936, exposure to increased air pollution in young through middle adulthood was associated with an increase in Horvath’s biological age years later (Baranyi et al., 2022). Pollution-related studies also show that while urine concentrations of arsenic, cadmium, lead, manganese and mercury did not influence age acceleration among 48 studied members of the NAS population, blood levels of vanadium, cobalt, zinc and barium caused negative age acceleration, and nickel and arsenic levels were associated with positive age acceleration in a Chinese cohort (Nwanaji-Enwerem et al., 2020; Xiao L. et al., 2021). An American study found that among a population highly exposed to polybrominated biphenyl also exhibited accelerated aging (Curtis et al., 2019).

Reproductive health has been connected to biological aging. Studies have found positive associations with Horvath biological age acceleration and age at menarche, age at menopause, time since menopause or surgically induced menopause (bilateral oophorectomy) (Chen M. et al., 2019; Levine et al., 2016). However, no association between Horvath’s biological age and pre-eclampsia diagnosis was reported (Pruszkowska-Przybylska et al., 2022). Epigenetic age association was found to increase slightly per live birth, and a woman’s age at first birth was inversely associated with age acceleration (Kresovich et al., 2019). Gestational impacts to biological age can also persist throughout life: in a study of 92 Canadian adults, men who had been born at an extremely low birth weight exhibited an advanced epigenetic age relative to men who were born at normal birth weight, or women regardless of birth weight (Van Lieshout et al., 2021). A small additional study found a similar result linking extremely low birth weight to later advanced epigenetic age, but a third paper published in 2022 found no association (Kuzawa et al., 2022; Mathewson et al., 2021).

Brain-related and mental health also exhibits associations with biological age. Data from the San Antonio Family Study found that global white matter index was negatively correlated with age acceleration. An MRI study of 79 participants found that cortical thickness decreased with advancing combined Horvath/Hannum biological age (Proskovec et al., 2020). A study of an extended Mexican-American pedigree found an association with reductions in white matter integrity and accelerated Horvath age, with evidence that this is due to underlying genetic influences (Hodgson et al., 2017). However, the findings associating cognitive capacity with changes in biological age using Horvath’s pan-tissue clock have been mixed. Work from the HANDLS and Middle-Aged Danish Twin Study cohorts found no association with cognitive function and Horvath biological age (Beydoun et al., 2020; Starnawska et al., 2017). Conversely, a small study of 68 cognitively healthy adults found that selective attention performance was better predicted by a combined Horvath/Hannum biological age than by chronological age (Wiesman et al., 2020). The VITAL-DEP study found an inverse association with objective cognitive score and accelerated biological aging among 23 participants (Vyas et al., 2019). In their study of 29 participants, Cruz-Almeida and colleagues found lower fluid cognition associated with greater biological age (Cruz-Almeida et al., 2019). They also observed that greater emotional stability, conscientiousness, and lower extraversion associated with a younger epigenetic age, while lower heat and pressure pain thresholds at the trapezius was associated with a greater epigenetic age. However, a number of studies report no association between Horvath’s biological age and cognitive decline or decline in mental facility (O’Shea et al., 2022; Shadyab et al., 2022; Sugden et al., 2022; Vetter et al., 2022a). Conflicting results were found in studies of schizophrenic individuals: a 2020 study found that male and female schizophrenics exhibited increased age acceleration, and that males taking clozapine alone or in conjunction with antipsychotic medication partially ameliorated that acceleration, while a 2018 study had found that in post-mortem brain samples there was no difference in biological age relative to controls, and blood samples from male schizophrenics exhibited reduced age acceleration, with no corresponding effect seen in female schizophrenics (Higgins-Chen et al., 2020; McKinney et al., 2018). A 2021 study found no significant difference between controls and schizophrenic patients in terms of biological age, but did report Horvath age acceleration correlating with psychotic score (Dada et al., 2021). The absence of a significant association between Horvath’s biological age and hypersexual disorder was reported in a 2022 paper describing 60 patients and 33 healthy controls (Boström et al., 2022).

No associations between autism diagnosis or ADHD genetic burden have been reported (Arpawong et al., 2023; Okazaki et al., 2022). While two studies observed an association with Horvath age acceleration and post-traumatic stress disorder, a study of caregivers found no increase in biological age associated with the stress of caring for a child with autism versus a neurotypical child (Robinson et al., 2020; Hamlat et al., 2021; Wang et al., 2022). A 2019 study found an association with increased age acceleration and anxiety, but not with depression (Vyas et al., 2019). Mental health interventions appear to be beneficial: a study of meditators found that each year of regular meditative practice was associated with a decrease in age acceleration among older, but not younger, meditators, and a randomized controlled trial including relaxation activities resulted in younger epigenetic ages relative to controls following 8 weeks of practice (Chaix et al., 2017; Fitzgerald et al., 2021). Studies on sleep quality have revealed mixed results. A study involving participants from the WHI found no impact of sleep disturbance on age acceleration, while a study combining data from the MESA and Framingham Heart Study (FHS) populations found that most disordered breathing traits were associated with epigenetic age acceleration in men only, although the associations of sex and sleep disturbances were not as clear with further statistical analysis (Carroll et al., 2017; Li et al., 2019).

#### 3.3.2 Horvath’s skin and blood clock

In contrast to the widespread use of Horvath’s pan-tissue clock, Horvath’s skin and blood clock, developed in 2018 using 391 CpGs, has yet to achieve similar levels of adoption (Horvath et al., 2018). We identified eleven papers that make use of the skin and blood clock for this review. Treatment of skin biopsies with senomorphic rapamycin revealed a biological age increase according to the skin and blood clock, contrary to the finding by a newly described skin-specific epigenetic clock (Boroni et al., 2020). The Horvath skin and blood clock reported the equivalent of 25 years of aging over a 152 days long course of growth of cultured fibroblasts, including during senescence (Sturm G et al., 2019). The study also found that fibroblasts grown under hyperglycemic conditions exhibited 3.4 years elevation in predicted biological age. However, a study of type I diabetics found that while the skin and blood clock did correlate with chronological aging, it predicted lower biological age in those participants, and no association with risk of cardiovascular disease, diabetic retinopathy, or neuropathy (Roshandel et al., 2020). Lower skin and blood clock age was associated with increased levels of physical activity, and higher age was associated with increasing BMI. In a study of the HRS population, white study participants were found to be biologically older than other participants, while for non-white participants, there was an association of experience of discrimination with elevated epigenetic age (Beydoun et al., 2022). No associations between the skin and blood clock were identified for substance abuse disorder, early ovarian aging, weight loss and/or metformin therapies, autism diagnosis, pre-eclampsia, socioeconomic status, and survival of head and neck cancers (Cabrera-Mendoza et al., 2023; Christensen et al., 2021; Nwanaji-Enwerem et al., 2021a; Okazaki et al., 2022; Pruszkowska-Przybylska et al., 2022; Schmitz et al., 2022; Xiao C. et al., 2021).

#### 3.3.3 Hannum’s clock

Hannum’s epigenetic clock was the first multi-CpG clock published, and relies on the methylation signatures of 71 CpG sites (Hannum et al., 2013). The clock was developed using blood methylation data exclusively, rather than employing a multi-tissue approach to estimating biological age. Of the 75 papers reviewed here that make use of the Hannum clock, all of them that reported it showed a positive correlation with chronological age; further, all that reported a correlation found a positive association with the biological age predicted by Horvath’s pan-tissue clock as well (Belsky et al., 2018a; Chen M. et al., 2019; Dada et al., 2021; Dugué et al., 2018; Li et al., 2015; Marioni et al., 2015; Roetker et al., 2018; Soriano-Tárraga et al., 2016). Hannum epigenetic age was not found to associate with facial aging (Marioni et al., 2018). There have been sex-specific differences reported, with men exhibiting a higher Hannum age than women (Bao et al., 2022; Baranyi et al., 2022; Beydoun et al., 2022; Dugué et al., 2018; Faul et al., 2023; Gensous et al., 2020; Kuzawa et al., 2022; Li et al., 2020; Martin et al., 2021; Sugden et al., 2022; Vetter et al., 2022b). As observed with the Horvath pan-tissue clock, white study participants exhibited a higher Hannum age acceleration than did African American study participants (Tajuddin et al., 2019). Hannum clock-measured biological age has an estimated heritability of 42%, and is found to be significantly correlated in monozygotic twin pairs as compared to non-twin sibling pairs (Li et al., 2015; Marioni et al., 2015). In a study of middle-aged Danish twins, the Hannum measure was found to overestimate the age of study participants (Starnawska et al., 2017). The Hannum clock correlates strongly when measured on the Illumina Infinium Human Methylation 450 k BeadChip and the Infinium MethylationEPIC BeadChip (McEwen et al., 2018). Sturm and colleagues found that Hannum’s clock predicted a linear age increase in cultured fibroblasts up until the senescence phase, when the clock ceased to progress (Sturm et al., 2019).

Studies have found elevated Hannum age or acceleration associated with increased risk of all-cause mortality (Faul et al., 2023; Li et al., 2020; Marioni et al., 2015). Among patients with head and neck cancer, increased morbidity was associated with elevated Hannum age (Xiao C. et al., 2021). In studies of ischemic stroke survivors, Hannum age acceleration was observed in survivors versus controls, with stroke survivors exhibiting an age 2.5 years older (Soriano-Tárraga et al., 2017). Elevated Hannum biological age, but not Horvath pan-tissue age, was associated with poorer outcomes 3 months following stroke (Soriano-Tárraga et al., 2017). Increased levels of cardiovascular dysregulation markers were associated with higher Hannum age acceleration in women only (McCrory et al., 2020). Increased risk for cardiovascular disease and mortality in the first and second decades following initial observation were all associated with Hannum age acceleration in a study of middle-aged African Americans (Roetker et al., 2018). However, in a study of Swedish senior citizens, no relationship between Hannum age and cardiovascular disease was detected (Lind L. et al., 2018). In unadjusted models, Roberts and colleagues report a link between Hannum age acceleration and atrial fibrillation (Roberts et al., 2021). Decline in FEV1, a marker of pulmonary health, was associated with Hannum age among female but not male participants of the SAPALDIA and European Community Respiratory Health Survey (ECRHS) cohorts (Rezwan et al., 2020). Twins exhibiting higher Hannum age acceleration were more than twice as likely to die earlier than the lower Hannum aged twin (Christiansen et al., 2016). Work with the Irish Longitudinal Study on Aging population found an association between increased Hannum age acceleration and the likelihood of taking five or more medications daily, a proxy feature of multimorbidity (McCrory et al., 2021). Members of the Lothian Birth Cohort of 1936 were significantly more likely to be frail when they exhibited Hannum age acceleration (Gale et al., 2018b; Gale et al., 2018a). A study of postmenopausal women found that the odds of maintaining mobility at age 90 worsened with increased biological age (Jain et al., 2022). Conversely, other studies have found no association with Hannum biological age and impairment of activities of daily life or physical decline (Graf et al., 2022; Vetter et al., 2022a). Belsky and colleagues report Hannum age acceleration associated with most of the markers of decreased health span measured in their study of 1,037 members of the Dunedin Study cohort (Belsky et al., 2018b). In terms of physical activity, results are mixed: a study of veterans with COPD revealed an improvement in Hannum biological age with an increase in exercise capacity, but other studies report no association with levels of physical activity (Fox et al., 2023; Spartano et al., 2023; Wan et al., 2021).

A number of physiological features associate with changes in Hannum biological age. Chiefly among them is BMI and related features including waist to hip ratio and waist circumference (Dugué et al., 2018; Fiorito et al., 2019; Kresovich et al., 2021b). Diabetes and gestational diabetes diagnoses were also associated with Hannum age acceleration (Kresovich et al., 2019; Roetker et al., 2018). Elevated HbA1C was associated with increased Hannum age acceleration, particularly in men, and metabolic dysregulation biomarkers were associated with increased Hannum biological age in both sexes (McCrory et al., 2020). Features of reproductive health associate with the Hannum clock: a woman’s age at first live birth was associated with decreased Hannum age acceleration, while a small age acceleration was associated with each additional live birth (Chen M. et al., 2019; Kresovich et al., 2019). There was no association observed with pre-eclampsia during pregnancy, nor with maternal energy reserves as represented by arm fat (Kuzawa et al., 2022; Pruszkowska-Przybylska et al., 2022). Further, there was no association detected between Hannum’s biological age later in life and birth weight (Kuzawa et al., 2022).

Work with the Melbourne Collaborative Cohort found that increased dietary fruit intake decreased Hannum age acceleration, but other studies analyzing the effects of adherence to a Mediterranean diet did not improve Hannum age (Dugué et al., 2018; Gensous et al., 2020; Kresovich et al., 2022). Weight loss regimens, including the CALERIE intervention program and a combination therapy including metformin, did not impact Hannum epigenetic age acceleration (Nwanaji-Enwerem et al., 2021a; Waziry et al., 2023). Dietary supplementation of vitamin D was also found to be associated with improved Hannum age, although serum levels of 25-hydroxyvitamin D were not; a later study did not replicate the finding linking vitamin D levels to epigenetic age (Chen L. et al., 2019; Vetter et al., 2022b). Beach and colleagues found in a study of the Family and Community Health Studies (FACHS) cohort that 55 of the 71 CpGs of the Hannum clock associate significantly with alcohol consumption, while 33 CpG sites respond significantly to smoking; while smoking always led to Hannum age acceleration, moderate levels of alcohol consumption reduced Hannum age, while very low and high levels of alcohol consumption were accelerative (Beach et al., 2015). A later study of the Sister Study population reaffirmed that current alcohol consumption was associated with increased Hannum epigenetic age (Kresovich et al., 2021a). Several other studies have noted the association between current or prior smoking and Hannum age acceleration (Dugué et al., 2018; Fiorito et al., 2019; Lei et al., 2017; Roetker et al., 2018). In a small study of 22 current smokers, successful smoking cessation led to a Hannum age deceleration (Lei et al., 2017). A study of Finnish mixed-gender twin pairs indicated that, while the male twin was biologically older, this effect was largely mediated by an increased likelihood to be a smoker relative to his sister (Kankaanpää et al., 2022b). In terms of air pollution and its impact on biological age, results are mixed: one study found no association with historical air pollution levels and Hannum age acceleration later in life, while a separate, smaller study identified a detrimental impact of immediate exposure to air pollution prior to testing (Baranyi et al., 2022; Gao et al., 2022a). A study of a population exposed to polybrominated biphenyl found that Hannum age acceleration associated with increasing exposure (Curtis et al., 2019). Similarly, exposure to certain organochlorine pesticides was associated with accelerated Hannum age in Swedish senior citizens (Lind P. M. et al., 2018). A study of long-term residents of Guangxi, China found that serum levels of vanadium, cobalt, zinc and barium associated with a decrease in Hannum age acceleration, while levels of nickel and arsenic accelerated Hannum age (Xiao L. et al., 2021).

Studies of mental health have revealed associations with the Hannum epigenetic clock. In a comparison of schizophrenic and control individuals, Hannum age acceleration was observed in schizophrenics (Dada et al., 2021). Male, but not female, schizophrenia patients taking clozapine showed a decrease in Hannum biological age relative to those not taking clozapine (Higgins-Chen et al., 2020). Anxiety, but not depression, was associated with Hannum age acceleration in a large 2020 study, but a later study of twins revealed a higher Beck Depression Inventory II score associated with elevated Hannum biological age (Liu et al., 2022; Robinson et al., 2020). In men, accelerated Hannum aging was associated with a faster decline in memory, visuoconstructive ability, attention and processing speed, but a previous study in a larger population found that Hannum biological age was not a good indicator of cognitive change in the middle aged (Beydoun et al., 2020; Starnawska et al., 2017). Additional studies revealed no apparent association between cognitive performance and Hannum’s clock (O’Shea et al., 2022; Shadyab et al., 2022; Vetter et al., 2022a). However, among people living with HIV, decreasing neural performance was associated with elevated biological age (Schantell et al., 2022). Autism, hypersexual disorder, and substance abuse disorder showed no association with Hannum’s clock, but a twin study revealed that twins with PTSD were biologically older than their unaffected sibling (Boström et al., 2022; Cabrera-Mendoza et al., 2023; Okazaki et al., 2022; Wang et al., 2022).

Low income and financial pressure have been associated with accelerated biological aging (Lei et al., 2019; Robinson et al., 2020; Simons et al., 2016). Similarly, neighborhood quality has been associated with Hannum age acceleration (Lei et al., 2019; Martin et al., 2021). Socioeconomic status results have been mixed: some studies report no significance, while others report that reduced childhood social class or economic status is associated with accelerated Hannum age, although one 2023 study reports an inverse finding for childhood financial hardship resulting in decelerated aging (Bao et al., 2022; Faul et al., 2023; George et al., 2021; Schmitz et al., 2022). A study of the HRS population reported the absence of an association with the experience of discrimination, and a later study of the same population detected increased familial contact associated with a lower Hannum age (Beydoun et al., 2022; Hillmann et al., 2023). Finally, Hannum biological age has been shown to associate negatively with educational attainment (Fiorito et al., 2019; Roetker et al., 2018).

#### 3.3.4 PhenoAge

In 2018 Levine and colleagues published their second generation epigenetic clock based on 513 CpGs, referred to as PhenoAge (Levine et al., 2018). This clock was developed using DNA methylation data from whole blood, and is designed to collect methylation status not only from chronological age-linked CpG sites, but also sites that are responsive to changes in physiological status. PhenoAge does correlate well with chronological age in blood and other cell types, and among the 70 papers reviewed for their use of PhenoAge, all that reported it found an association with chronological age. Furthermore, PhenoAge shows correlation with first generation clocks (Cabrera-Mendoza et al., 2023; Chen M. et al., 2019; Dolcini et al., 2020; Hamlat et al., 2021; Nwanaji-Enwerem et al., 2020; Roshandel et al., 2020; Wang et al., 2022). PhenoAge also shows correlation with other second and third generation clocks, such as DunedinPoAm and GrimAge (Belsky et al., 2020; Cabrera-Mendoza et al., 2023; Dolcini et al., 2020; Nwanaji-Enwerem et al., 2020; Roshandel et al., 2020; Wang et al., 2022). Clocks not extensively based on DNA methylation, such as measures of allostatic load and metabolomic metrics, show association with PhenoAge (McCrory et al., 2020; Robinson et al., 2020). It also correlates negatively with mtDNA copy number, but studies considering telomere length and PhenoAge are mixed (Cabrera-Mendoza et al., 2023; Dolcini et al., 2020; Hastings et al., 2019; Seki et al., 2023). Contrary to findings in other clocks, two studies report finding lower PhenoAge in men relative to women; Li and colleagues found that increased PhenoAge was associated with increased mortality risk, with the effect much stronger in women than in men (Li et al., 2020; Kuzawa et al., 2022; Martin et al., 2021). However the majority of studies that report a sex-based difference find women with a younger PhenoAge than men (Avila-Rieger et al., 2022; Baranyi et al., 2022; Faul et al., 2023; Kankaanpää et al., 2022b; O’Shea et al., 2022; Sugden et al., 2022; Vetter et al., 2022b). The results of ethnicity-based analyses are mixed: five studies report ethnicity-based PhenoAge, with one finding Hispanics age at a slower rate than non-Hispanics, two reporting no association with race or ethnicity among study participants, and two reporting higher PhenoAge acceleration among Black participants as compared to whites or Hispanics (Avila-Rieger et al., 2022; Beydoun et al., 2022; Faul et al., 2023; Graf et al., 2022; Kresovich et al., 2023). A study of cultured fibroblasts found that PhenoAge increased linearly over the growth phase of the cells, but ceased to increase at senescence (Sturm et al., 2019). Two of the studies reviewed here found that PhenoAge systematically underestimates chronological age, one examining biological age among HIV patients prior to and during antiretroviral therapy, and one examining a large cohort of older adults (Esteban-Cantos et al., 2021; Shireby et al., 2020). However, PhenoAge does associate with markers of multimorbidity, limitations in activities of daily life, cognitive function, and increased risk of mortality (Crimmins et al., 2021; Gao et al., 2019; Graf et al., 2022; Hastings et al., 2019; Li et al., 2020). A study of postmenopausal women linked elevated PhenoAge acceleration with a decrease in the likelihood of surviving to age 90 with intact mobility and cognitive function (Jain et al., 2022). In contrast, a study of 490 participants in the Irish Longitudinal Study of Aging found no association between PhenoAge and any of the physical or cognitive outcomes measured in that study, and a study of the BASE II population had similar non-significant findings (McCrory et al., 2021; Vetter et al., 2022a). Both poorer initial performance and accelerated decline in memory function tests are associated with worsening PhenoAge acceleration, although the effect is partially mediated by socioeconomic status (Avila-Rieger et al., 2022). Additional studies analyzed find no association with cognitive functioning or dementia with PhenoAge acceleration (O’Shea et al., 2022; Shadyab et al., 2022; Sugden et al., 2022).

Individual physical parameters associate with changes in PhenoAge. Sleep quality in terms of a higher apnea/hypopnea index and arousal index associated with PhenoAge acceleration in women but not in men in the MESA population, but the inverse sex association was found in the FHS population (Li et al., 2019). In the UK Biobank population, an early chronotype, and achieving normal sleep duration were associated with a decelerated PhenoAge (Gao et al., 2022b). That study also observed that daytime sleepiness and difficulty rising was associated with an accelerated PhenoAge. Serum levels of vitamin B6 negatively associate, while serum folate levels positively associate with PhenoAge; vitamin D supplementation did not influence PhenoAge in either healthy or vitamin D deficient study participants in a separate study (Nwanaji-Enwerem et al., 2021b; Vetter et al., 2022b). As with other clocks described here, BMI associated positively with increasing PhenoAge, as do waist to hip ratio and waist circumference (Cao et al., 2023; Chen M. et al., 2019; Faul et al., 2023; Fiorito et al., 2019; Kankaanpää et al., 2022b; Kresovich et al., 2021b; Kresovich et al., 2023; Xiao et al., 2021). Becoming obese resulted in age acceleration in a study of the NHANES population (Cao et al., 2023). Comparing men and women, a Finnish twin study found that the tendency for males to exhibit a higher PhenoAge than females is partially modulated by higher BMI observed in male study participants (Kankaanpää et al., 2022b). An intervention trial comparing conventional weight loss with metformin treatment or combination therapy found no improvement in PhenoAge acceleration among overweight and obese female study participants; similarly, dietary intervention in the CALERIE trial did not affect PhenoAge (Nwanaji-Enwerem et al., 2021a; Waziry et al., 2023). Conversely, the Sister Study population found that adherence to a Mediterranean or DASH diet was associated with lower PhenoAge, particularly among women with low physical activity (Kresovich et al., 2022). Higher levels of daily physical activity, including increased 6 minute walk distance and increases in grip strength, were reported to associate with decreased PhenoAge (Fox et al., 2023; Loh et al., 2023; Seki et al., 2023). In a longitudinal study of pregnant women, it was found that increases in BMI during pregnancy were associated with lower post-birth PhenoAge, but that having no BMI decrease in the year post-birth was associated with a higher post-birth PhenoAge (Ross et al., 2020). Diagnosis of pre-eclampsia resulted in an elevated PhenoAge as compared to normotensive pregnant women (Pruszkowska-Przybylska et al., 2022). Self-reported abnormal glucose tests during pregnancy and diagnosis of gestational diabetes were found to be associated with increased PhenoAge, but in a cell culture study, growth in hyperglycemic conditions did not lead to a PhenoAge acceleration (Kresovich et al., 2019; Sturm et al., 2019). Among women with early ovarian aging, no difference in PhenoAge was detected relative to women with normal ovarian aging (Christensen et al., 2021). Additionally, other features of reproductive health associate with PhenoAge: PhenoAge acceleration occurs slightly after each live birth, but was inversely correlated with a woman’s age at first live birth (Kresovich et al., 2019). There was no observed association with age at menarche or other features of pubertal timing (Hamlat et al., 2021). Among males only, birth weight was inversely associated with PhenoAge later in life (Kuzawa et al., 2022).

Chronic disease is associated with higher PhenoAge. Multiple sclerosis patients were found to have a higher PhenoAge independent of BMI or smoking status (Theodoropoulou et al., 2019). Studies of HIV positive individuals reveal that low CD4^+^ cell counts show significant PhenoAge acceleration, and antiretroviral therapy leads to a decrease in PhenoAge (Esteban-Cantos et al., 2021). In schizophrenia, a higher PhenoAge is observed in patients as compared to controls, but unlike the results found for the Horvath pan-tissue and Hannum clocks, no decrease in PhenoAge was observed among patients on clozapine or clozapine in conjunction with antipsychotic medications (Higgins-Chen et al., 2020). No association was observed with diagnosis of autism or hypersexual disorder, but a higher Beck Depression Inventory II score was associated with an accelerated PhenoAge (Boström et al., 2022; Liu et al., 2022; Okazaki et al., 2022). Among individuals with substance abuse disorder, an elevated PhenoAge was detected relative to controls in blood samples, but not in study of 49 post-mortem brain samples (Cabrera-Mendoza et al., 2023). A twin study revealed that twins with post-traumatic stress disorder have an elevated PhenoAge relative to their healthy twin (Wang et al., 2022). Roshandel and colleagues found in a study of type I diabetics that declining kidney function was associated with increased PhenoAge, but other diabetic complications were not (Roshandel et al., 2020). Metabolic dysregulation, a subtype of dysregulation under the allostatic load measurement, was found to be correlated with increased PhenoAge, while cardiovascular dysregulation was only significant in women (McCrory et al., 2020). Hypertension and atrial fibrillation been observed in association with increased PhenoAge acceleration (Roberts et al., 2021; Robinson et al., 2020). In a small study, chemotherapy treatment did not associate with increased PhenoAge acceleration, while for radiation therapy, increased age acceleration at 6 and 12 months post-therapy were associated with worse overall survival and progression free survival (Loh et al., 2023; Xiao et al., 2021).

Environmental and lifestyle exposures also influence PhenoAge, in particular smoking and heavy alcohol consumption (Bøstrand et al., 2022; Chen M. et al., 2019; Faul et al., 2023; Fiorito et al., 2019; Kresovich et al., 2023; Robinson et al., 2020; Simons et al., 2022a; Simons et al., 2022b). Work on the Sister Study population did not confirm an association with alcohol consumption and accelerated PhenoAge, however (Kresovich et al., 2021a). A Finnish twin study found that while male twins were more likely to have a higher PhenoAge than their sisters, this affect was modulated by unhealthy lifestyle habits including smoking and alcohol use (Kankaanpää et al., 2022b). Study of those exposed to polybrominated biphenyl found PhenoAge increased with increased exposure (Curtis et al., 2019). Urinary metal concentrations measured in participants of the NAS was found to associate with PhenoAge acceleration, in particular levels of manganese alone, or in a mixture model a combination of arsenic, cadmium, lead, manganese and mercury (Nwanaji-Enwerem et al., 2020). Air pollution was not found as significant in a study of the Lothian Birth Cohort of 1936, but work with the UK Biobank population did find an association with higher ${\text{PM}}_{2.5}$ and atmospheric ${\text{NO}}_{2}$ levels and increased PhenoAge (Baranyi et al., 2022; Gao et al., 2022b). There was no impact detected from immediate air pollution exposure prior to testing in a population of 26 Chinese students, however (Gao et al., 2022a). Beyond exposure to pollution, neighborhood quality was found to have a significant association with PhenoAge in women; a later study had similar findings, with no gender effect (Lei et al., 2022a; Martin et al., 2021). Income and educational attainment are both negatively associated with PhenoAge (Bao et al., 2022; Fiorito et al., 2019; George et al., 2021; Kresovich et al., 2023; Robinson et al., 2020). Other measures of socioeconomic status, both during childhood and adulthood, associated with PhenoAge (Bao et al., 2022; Faul et al., 2023; George et al., 2021; Schmitz et al., 2022). Childhood adverse experiences have been studied in relation to accelerated PhenoAge later in life, although the findings have been limited: one study detected an association with abusive, but not neglectful, childhood maltreatment associated with accelerated biological aging, but other studies report no association with adverse experiences or childhood police encounters (Beydoun et al., 2022; Das, 2022; Joshi et al., 2023; Rampersaud et al., 2022). No association was found between perceived discrimination or social isolation and elevated PhenoAge (Beydoun et al., 2022; Das, 2022; Hillmann et al., 2023).

#### 3.3.5 GrimAge

GrimAge is a second generation epigenetic clock that includes CpG methylation estimates of seven key physiological risk and stress-related plasma proteins, as well as a CpG-based indicator of total smoking pack years (Lu et al., 2019). It assesses methylation at 1,030 CpG sites and is especially accurate at estimating time to mortality. A relatively recently developed clock, 65 papers studied in this review employed GrimAge, and for the papers that reported it, GrimAge was found to correlate with chronological age. Three studies reported a significant correlation in predictions of GrimAge with both Horvath’s pan-tissue and PhenoAge (Nwanaji-Enwerem et al., 2020; Nwanaji-Enwerem et al., 2021a; Roshandel et al., 2020). An additional study found no relationship between mtDNA copy number and GrimAge (Dolcini et al., 2020). One study reviewed here found that GrimAge tended to overestimate age (Esteban-Cantos et al., 2021). Elevated GrimAge was found in men relative to women, and lower in white individuals as compared to other ethnicities (Arpawong et al., 2023; Avila-Rieger J et al., 2022; Baranyi G et al., 2022; Graf GH et al., 2022; Hamlat et al., 2021; Kankaanpää A et al., 2022b; Kresovich JK et al., 2023; Kuzawa et al., 2022; McLachlan et al., 2020; O’Shea DM et al., 2022; O’Shea DM and Galvin JE, 2023; Peterson et al., 2023; Simons et al., 2021; Simons et al., 2022a; Sugden K et al., 2022; Vetter VM et al., 2022b). GrimAge and GrimAge acceleration strongly associate with increased risk of mortality: two studies associated a 39% and 91% increase in mortality associated with one standard deviation increase in GrimAge, the former study observing a stronger effect in women (Li et al., 2020; McCrory et al., 2021). Accelerated GrimAge was associated with decreased facility in the activities of daily life, with poorer health span-related measures and with decreased probability of achieving age 90 with intact motility (Faul et al., 2023; Graf et al., 2022; Jain et al., 2022). Conversely, a 2022 study did not find GrimAge as a good marker of either physical or mental health deterioriation (Vetter et al., 2022a). Increased GrimAge is associated with diagnoses of chronic disease as well as with polypharmacy (McCrory et al., 2021; McLachlan et al., 2020). Development of diabetes in patients with obesity and/or hyperglycemia, as well as the incidence of three key diabetic complications, were associated with elevated GrimAge acceleration (Kim et al., 2021; Roshandel et al., 2020). Increased likelihood of atrial fibrillation was found to be associated with elevated GrimAge (Roberts et al., 2021). Studies of lung health in a large, combined cohort found an association of GrimAge with reduced FEV1, FVC, as well as the ratio of FEV1/FVC (Rezwan et al., 2020). There was no association of GrimAge and pre-eclampsia compared to normotensive pregnancy identified in a 2022 study (Pruszkowska-Przybylska et al., 2022). Studies on GrimAge in other chronic diseases are limited. Higgens-Chen and colleagues found GrimAge acceleration in schizophrenic patients versus controls (Higgins-Chen et al., 2020). Increased ADHD polygenic score is associated with an accelerated GrimAge; in a study of high functioning autistics, only one component of GrimAge (DNAmPAI-1) was significantly associated, with GrimAge overall having insignificant associations (Arpawong et al., 2023; Okazaki et al., 2022). Patients diagnosed with hypersexual disorder did not exhibit elevated GrimAge relative to healthy controls (Boström et al., 2022). In a study of HIV positive individuals, significant GrimAge acceleration was observed among those with a high viral load, which improved following antiretroviral therapy (Esteban-Cantos et al., 2021).

Some studies investigated various facets of mental and neurological health and biological aging. Findings related to depression are mixed: a 2023 report identifies an association with increased GrimAge that is not supported by an earlier report finding no connection between depression severity and GrimAge (Faul et al., 2023; Liu et al., 2022). Three studies investigating trauma-exposed and PTSD-diagnosed veterans found differing patterns of association as well: a 2021 study affirmed a link between GrimAge acceleration and PTSD diagnosis and severity, while a 2022 study found the PTSD link only significant among younger veterans, and a 2023 study found a link with PTSD but no improvement following treatment (Hawn et al., 2022; Katrinli et al., 2023; Yang et al., 2021). The 2022 study also observed that younger veterans had significant association between cognitive disinhibition and poorer memory with GrimAge, while inflammatory and neuropathological traits were associated regardless of chronological age. When comparing nonpathological differences in cognitive ability, it was found that women possibly exhibited a younger GrimAge than men due to differences in those abilities (O’Shea et al., 2022). A further study of GrimAge in American veterans uncovered links with educational attainment, marital status, combat history, accumulated past traumatic experiences and substance abuse disorder with GrimAge, while increases in physical activity frequency, improvements in sleep quality, and a predisposition towards openness and gratitude were all found to be protective (Tamman et al., 2023). In a study of APOE ε 4 status, it was found that there was an interaction with verbal memory in women only and GrimAge acceleration (O’Shea and Galvin, 2023). Poorer initial memory performance and a steeper decline in performance both associated with accelerated GrimAge, a finding that was partially mediated by socioeconomic status (Avila-Rieger et al., 2022). There have been no studies positively identifying an association with GrimAge acceleration and diagnosis of either mild cognitive impairment (MCI) or dementia, however (Shadyab et al., 2022; Sugden et al., 2022).

Studies concerning the relationship between BMI and GrimAge are mixed. In a study of 2,758 non-Hispanic, middle-aged white women, GrimAge was found to be associated with BMI, waist to hip ratio, and waist circumference, while a smaller study found no association with BMI (Kresovich et al., 2021b; McCrory et al., 2021). Later studies are more decisive, finding a positive association between BMI and accelerated GrimAge (Faul et al., 2023; Kankaanpää et al., 2022a; Yang et al., 2021). Dietary interventions have also been mixed: a 2023 study of the CALERIE intervention found no significant effect, while a 2022 investigation found both the DASH and Mediterranean diets associated with decreased GrimAge (Kresovich et al., 2022; Waziry et al., 2023). A trial involving metformin, conventional weight loss, or combination therapy in overweight and obese breast cancer survivors did not find an association with GrimAge, however (Nwanaji-Enwerem et al., 2021a). In a study of pregnant women, there was a significant decrease in GrimAge measured at post-birth relative to during pregnancy, and that increases in BMI during pregnancy were associated with a lower post-birth GrimAge, but that having no decrease in BMI in the year post-birth was associated with a higher GrimAge (Ross et al., 2020). Birth weight is not reported to be associated with GrimAge (Kuzawa et al., 2022). Nwanaji-Enwerem and colleagues found that levels of vitamin B6 are associated with decreased GrimAge, while folate levels are associated with elevated GrimAge (Nwanaji-Enwerem et al., 2021b). In a study of the BASE II population, vitamin D levels were found to be inversely associated with GrimAge (Vetter et al., 2022b). History of and current smoking, as well as elevated alcohol consumption, were associated with increased GrimAge (Bøstrand et al., 2022; Faul et al., 2023; Kankaanpää et al., 2022b; Katrinli et al., 2023; Kresovich et al., 2021a; Li et al., 2020; McLachlan et al., 2020; Simons et al., 2021; Simons et al., 2022a; Simons et al., 2022b; Yang et al., 2021). However, in a relatively small study an association between GrimAge and substance abuse disorder in either blood or brain samples was not detected (Cabrera-Mendoza et al., 2023). Physical activity and elevated leisure activity were found to be associated with lower GrimAge acceleration among men in a Finnish twin study, but a more focused study comparing master rowers to age-matched sedentary controls did not find an effect of physical activity or prowess (Kankaanpää et al., 2022b; Seki et al., 2023). Other studies found the influence of increased physical activity on GrimAge deceleration, including higher daily step counts, improvements and ability in the 6 minute walk distance test, and hand grip strength were all reported (Fox et al., 2023; Loh et al., 2023; Peterson et al., 2023; Spartano et al., 2023; Wan et al., 2021). Joint pain, specifically low and high impact knee pain, conferred an older GrimAge biological age, and this partly mediated the effect of pain on balance performance (Peterson et al., 2022).

Socioeconomic features have been studied in conjunction with GrimAge. McLachlan and colleagues found elevated GrimAge with lower levels of educational attainment, lower social status, and psychosocial loss (McLachlan et al., 2020). Simons and colleagues found associations with educational attainment, household income, neighborhood disadvantage and cumulative disadvantage (Simons et al., 2021). Pollution levels have had mixed findings: a study of the Lothian Birth Cohort of 1936 found no influence on GrimAge from historical pollution levels, while a small study of Chinese students found that an increase in ${\text{PM}}_{2.5}$ prior to examination increases GrimAge (Baranyi et al., 2022; Gao et al., 2022a). Higher levels of early life abuse and trauma were associated with elevated GrimAge acceleration later in life (Hamlat et al., 2021). An additional study identified an association between cumulative adverse childhood experience score and GrimAge, but this finding was not corroborated by an earlier 2022 study of 81 participants (Joshi et al., 2023; Rampersaud et al., 2022). It was also found parental separation or divorce and childhood emotional abuse also correlated with accelerated GrimAge later in life (Joshi et al., 2023). Overall, both childhood and adult socioeconomic status associated with GrimAge, although the effects were found to be additive rather than multiplicative (George et al., 2021; Schmitz LL et al., 2022). Lower educational attainment and neighborhood disadvantage are both associated with accelerated GrimAge (Berg et al., 2022; George et al., 2021; Kresovich et al., 2023; Lei et al., 2022a). GrimAge is significantly associated with both childhood police encounters and previous incarceration (Berg et al., 2021; 2022; Das, 2022). Mental health considerations also factor into GrimAge and GrimAge acceleration: more symptoms of depression, as well as a negative attitude towards aging, are linked to GrimAge acceleration (McLachlan et al., 2020). Perceived everyday discrimination and cumulative disadvantage also associate with elevated GrimAge (Beydoun et al., 2022; Simons et al., 2022a; Simons et al., 2022b). Studies of African American youth found that changes in self control ability during late adolescence were associated with an improved GrimAge in early adulthood (Lei et al., 2022b). Higher levels of support or contact with friends or one’s children was associated with decelerated GrimAge (Hillmann et al., 2023).

#### 3.3.6 Weidner’s clock

In 2014, following the Hannum and Horvath pan-tissue epigenetic clocks, the Weidner clock was developed. This clock was originally developed to interrogate CpG methylation status via bisulphite pyrosequencing, rather than the then-costly microarray approach (Weidner et al., 2014). The Weidner clock is derived from the methylation status of 102 CpG sites, 99 of which are present on the Illumina Infinium Human Methylation 450 k BeadChip; many of the reviewed uses of the Weidner clock rely on microarrays and those 99 CpG sites rather than the complete 102 CpG clock. Weidner and colleagues distilled their clock down to a 3 CpG clock that was nearly as predictive as the complete 102 or 99 CpG sets; those three CpGs were associated with the genes PDE4C, ITGA2B and ASPA. Six papers reviewed here make use of the Weidner clock, and the three that assessed association with chronological age found a positive association (Florian et al., 2017; Lin et al., 2016; McEwen et al., 2017). One paper reported a weak correlation between the Weidner 99 CpG set and biological age as measured using 10 biomarkers and the Klemera-Doubal method, but none of the studies considered here reported any correlation with any of the other methylation-based clocks (Belsky et al., 2018a). A study of the combined Lothian Birth Cohorts of 1921 and 1936 found that per 5 year age acceleration according to the 99 CpG clock, there was an associated 11% increase in risk to mortality, however, only the CpG associated with PDE4C was significantly associated with survival (Lin et al., 2016). In the Lothian Birth Cohort of 1921 alone, there was no association between Weidner age and estimated face age, and a study of the Dunedin cohort found no association with any of the healthspan-related characteristics they measured (Marioni et al., 2018; Belsky et al., 2018b). No correlation was found with either the 99 or 3 CpG versions of the clock with experience of discrimination in a study of the HRS population (Beydoun et al., 2022).

#### 3.3.7 DunedinPoAm

The ‘Pace of Aging’ third generation clock, which measures the rate of physiological deterioration, was developed in 2015 by Belsky and colleagues, and is based on 18 biomarkers covering a variety of different organ systems, calculated using the Klemera-Doubal algorithm (Belsky et al., 2015; Klemera and Doubal, 2006). In that seminal paper, the authors compared their Pace of Aging clock against a 10 biomarker clock also calculated using the Klemera-Doubal method, and found they correlated positively and to a moderate degree.

In 2020, Belsky turned his attention to methylation markers of biological aging, and using Pace of Aging as the criterion, used elastic net regression to identify 46 CpG methylation sites associated with the Pace of Aging metric (Belsky et al., 2020). Interestingly, none of the 46 CpG sites selected overlap with those found in the Horvath pan-tissue, Hannum, or Levine PhenoAge clocks. This novel clock was called DunedinPoAm for the study group used to develop it. It was found that DunedinPoAm increases were associated with declines in physical and cognitive capability, and increases in physical limitations, facial aging and self-reporting of fair or poor health. Since its publication, usage of the DunedinPoAm clock has been steady. Sex-based difference in DunedinPoAm, where reported, has given mixed results (Avila-Rieger et al., 2022; Bao et al., 2022; Faul et al., 2023; Kuzawa et al., 2022; Sugden et al., 2022). Three studies report elevated DunedinPoAm epigenetic age in Black study participants than in other racial groups (Avila-Rieger et al., 2022; Faul et al., 2023; Kresovich et al., 2023; Shen et al., 2023). A twin study found weak correlation of DunedinPoAm with first generation clocks and only modest correlation with second generation epigenetic clocks (Kankaanpää et al., 2022a).

Lifestyle factors including obesity, smoking, alcohol consumption and low physical activity are all associated with elevated DunedinPoAm (Beach et al., 2022; Faul et al., 2023; Kankaanpää et al., 2022a; Kresovich et al., 2023; Simons et al., 2022a; Simons et al., 2022b). Conversely, dietary interventions such as the CALERIE trial resulted in improved biological age (Waziry et al., 2023). Birth weight was found to be inversely associated with DunedinPoAm age acceleration later in life (Kuzawa et al., 2022). Other physical parameters, such as post-intervention improvements in the 6 minute walk test distance and handgrip strength, were also associated with a decelerated DunedinPoAm in a study of 20 participants (Loh et al., 2023). Cognitive dysfunction is found to associate with accelerated aging: a 2022 study reported that dementia patients exhibited a higher biological age than did those with mild cognitive impairment, who in turn were biologically older than cognitively normal study participants (Sugden et al., 2022). That study also observed that the risk of dementia later in life as well as worse cognitive testing scores independent of diagnosis were both linked to elevated biological age. A study of the HRS population found that memory performance and rate of decline were associated with biological age, although the effect was partially mediated by socioeconomic status (Avila-Rieger et al., 2022). Applying DunedinPoAm to the NAS population found an association with chronic disease morbidity and mortality risk (Belsky et al., 2020). A 2022 study reported an association between elevated DunedinPoAm and functional impairment, mortality, activities of daily life limitations, chronic conditions, and self-rated health (Graf et al., 2022).

Associations between elevated biological age and elevated levels of childhood deprivation and victimization were found among the E-Risk study population (Belsky et al., 2020). Other studies affirmed the link between childhood socioeconomic status and DunedinPoAm aging later in life, as well as other childhood incidents including police encounters and general childhood instability (Bao et al., 2022; Das, 2022; Faul et al., 2023; Simons et al., 2022b). Educational attainment is inversely associated with biological age (Bao et al., 2022; Faul et al., 2023; Kresovich et al., 2023; Schmitz et al., 2022). In adulthood, socioeconomic status and household income and wealth, including changes in income, continue to associate with DunedinPoAm (Beach et al., 2022; Schmitz et al., 2022; Shen et al., 2023; Simons et al., 2022b). While cumulative disadvantage did associate, the experience of discrimination was not found to be associated with biological age (Beydoun et al., 2022; Schmitz et al., 2022; Simons et al., 2022a). Divorce was found to accelerate DunedinPoAm (Bao et al., 2022). Neighborhood features, including neighborhood disadvantage, are also linked to changes in biological age (Lei et al., 2022a; Schmitz et al., 2022). An increase in pollution exposure resulted in an elevated DunedinPoAm biological age (Gao et al., 2022a). Lastly, personal experience parameters, such as reductions in perceived loneliness and higher support and contact with friends, were related to lower levels of biological aging (Beach et al., 2022; Hillmann et al., 2023).

#### 3.3.8 Novel and specific clocks

There are a wide variety of lesser used epigenetic clocks as well as recently developed clocks, often designed for particular purposes. Five studies reviewed here made use of the seven CpG Vidal-Bralo clock, finding its correlation with chronological age and telomere length, as well as its increased precision in men relative to women (Banszerus et al., 2019; Higgins-Chen et al., 2020; Vetter et al., 2019; Vidal-Bralo et al., 2016). A pair of studies making use of the Vidal-Bralo clock in the BASE and BASE II populations found that while the seven CpG Vidal-Bralo did not correlate with morbidity, lung capacity, or subjective health, a modified eight CpG version of Vidal-Bralo did exhibit a weak association with cardiovascular health (Drewelies et al., 2022; Lemke et al., 2022). It was found that men with obstructive coronary artery disease exhibited an elevated biological age by the Vidal-Bralo clock (Banszerus et al., 2023). A 2020 study comparing the efficacy of 14 epigenetic clocks included Vidal-Bralo as well as the chronological age-trained Lin, Garagnani and Bocklandt clocks, the mortality-trained Zhang clock, and the mitotic division-trained MiAge and Yang clocks, and found that mortality-trained clocks showed accelerated aging in schizophrenics, while mitotic clocks showed decelerated aging, and chronologic age-trained clocks found deceleration among male schizophrenics taking clozapine (Higgins-Chen et al., 2020). The Lin clock was found to be significant for women with elevated depressive symptoms, but a second study found no link with that clock to socioeconomic status (Beydoun et al., 2022; Lin et al., 2016; Schmitz et al., 2022). The Yang EpiTOC clock was similarly found significant for depressive symptoms but was also found to be significant in the HRS but not MESA populations for association with socioeconomic status (Beydoun et al., 2022; Schmitz LL et al., 2022). The Zhang mortality-trained clock was additionally significant for depressive symptoms (Beydoun et al., 2022). The EpiTOC and MiAge clocks were examined in a study of obese and overweight breast cancer survivors undergoing weight loss, weight loss therapy supplemented with metformin, or metformin alone, but no significant difference in biological aging was detected after 6 months (Nwanaji-Enwerem et al., 2021a). The Wu epigenetic clock was the only clock among a variety tested to detect a biological age difference in pre-eclampsia affected pregnant women as compared to normotensive controls (Pruszkowska-Przybylska et al., 2022). In a study of French centenarians and supercentenarians and their offspring, four clocks of limited CpGs (all used 8 or fewer methylation sites) found that both the centenarians/supercentenarians and their offspring were younger than their chronological age by those clocks (Daunay et al., 2022).

Zhang and colleagues developed a 10 CpG mortality score, that while not correlated with chronological age, did correlate with mortality and frailness (Zhang et al., 2018). Methylation of a different 10 mortality-associated CpG sites were used to calculate an alternate “Mortality Risk Score,” which correlated with both Horvath’s pan-tissue and PhenoAge epigenetic clocks (Gao et al., 2019). Increases in this Mortality Risk Score were associated with increased all-cause mortality, fatal cardiovascular disease, and fatal cancer. The TruMe proprietary clock, which exhibits a good correlation with chronological age, was used to detect a decrease in biological age following 7 months of a pharmaceutical intervention in a trial involving 42 participants (Demidenko et al., 2021). The Aging 3.0 clock has also been used to study the HIV positive population; a 2023 study reports elevated age among cannabis but not tobacco smokers, as well as increased biological age in diabetics as compared to non-diabetics (Mamoshina et al., 2018; Peixoto et al., 2023).

Using a limited number of CpG sites and measurements of signal joint T cell receptor arrangement excision circles (sjTRECs), Paparazzo et al. were able to produce a blood based novel clock with a mean absolute error (MAE) of 4.43 years (Paparazzo et al., 2022).

Lastly, we address the novel, often purpose-focused clocks we examined for this review. Following the observation that many epigenetic clocks systematically underestimate the age of brain cortex samples, Shireby and colleagues developed a novel clock that more accurately assesses biological age in cortex samples (Shireby et al., 2020). This clock employed elastic net regression to select CpG methylation sites significantly associated with chronological age. Other novel clocks using elastic net regression include a skin-specific, a breast-specific, and a skeletal muscle clock (Boroni et al., 2020; Castle et al., 2020; Voisin et al., 2020). The skin-based clock was developed using 2,266 CpG sites, and was found to work well on both biopsied samples and tissue culture (Boroni et al., 2020). The breast-based clock relies on 286 CpG sites, and when tested on normal breast tissue highly correlates with chronological age (Castle et al., 2020). Breast tumor samples show biological aging acceleration regardless of HER2 and hormone receptor genotype, while for triple-negative breast tumors no apparent acceleration was observed. The skeletal muscle-derived clock relies on the contributions of 1975 CpG sites, and outperforms Horvath’s pan-tissue when it comes to estimating biological age from that tissue source (Voisin et al., 2020).

## 4 Discussion

**FIGURE 4:**
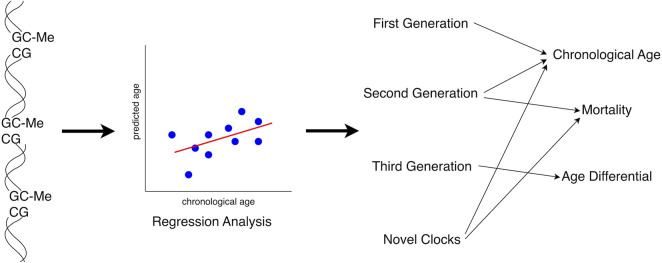
The relationship between CpG methylation, analysis typically by regression, and the different generations of clocks and novel clocks discussed in this paper with their trained output data. First generation clocks were trained on chronological age, GrimAge (a second generation clock) on mortality, and DunedinPoAm (a third generation clock) on the differential of predicted versus actual age.

The preceding Results section provides our mapping of domain knowledge available for distinct biological clocks and algorithms which make use of methylation-based epigenetic data. Many of the works here reviewed consider multiple methylation-based clocks, and we have resolved those findings into clock- and algorithm-specific sections to facilitate understanding. By categorizing experimental findings into these specific sections, we additionally enable comparisons between clocks and algorithms. Figure 4 summarizes the relationship between different clock generations and their input data, CpG methylation, with their output predictions, where biological age is represented as the differential between the chronological age and prediction in the case of first generation clocks, with the prediction of time to mortality in the case of GrimAge, and with the prediction of age differential for the third generation DunedinPoAm.

This work limits its focus to consideration of methylation-based measures of biological age and does not attempt to analyze the many non-methylation-based metrics for biological age determination. We have prepared a companion review article that correspondingly focuses on non-methylation measures of biological age (Ziesel et al., 2025) For comparison of these two broad categories, we direct readers to the combined version of these reviews (Ziesel et al., 2024).

While the past decade has witnessed the development of a number of epigenetic biological clocks, there remain outstanding questions on the relationship between epigenetic status and biological aging. Our scoping review methodology has identified gaps in knowledge, and we break these questions into three categories: questions regarding measurement, questions regarding biological aging itself and methylation measurement-specific questions including ethnicity- and sex-based differences.

### 4.1 Measurement questions

Among some of the papers reviewed here, we observe that biological clocks often concur regarding changes in biological aging rates in individuals. This leads one to question the function, whether responsive or generative, with respect to aging, and what distinct biological processes are implicated by those functions. The ability to differentiate between generative and responsive biological aging markers would open up possibilities in both intervention and diagnostic efforts. For aging responsive features, understanding the relationship between different environmental or health conditions has yet to be fully elucidated. Determining whether responsive features are acute or lagging responders, and persistent or transient responders to biological aging are a particularly relevant questions. Increased emphasis on longitudinal studies with repeated epigenetic measurements taken may help to ascertain the character of clock components.

### 4.2 Methylation-based measurement questions

Many measurements of biological age use CpG methylation and the focus on this feature has led to possible future research questions. Pre-eminently among those questions are: why do clocks not always agree in terms of the direction or magnitude of biological age differentials, and why is there a markedly limited overlap in the CpG sites employed by different methylation clocks? Is this limited overlap due to there being different markers of biological aging in different tissue types, or is there another explanation? Many methylation clocks show slower aging in female subjects, despite the relative acceleration of observed aging in female-specific tissues such as mature breast. Why does breast tissue typically display accelerated aging relative to the whole individual and, related, why would other clocks show decelerated aging in females relative to males? Further, if tissue-specific methylation clocks correctly measure aging in their respective tissues, does this imply that a generalized methylation clock is inherently limited for a heterogeneously aging organism? If so, does this imply that multi-tissue biomarkers are a more valid approach to the measurement of biological aging? Does this also imply that imprinted genes are also directly implicated in the process of biological aging under both normal and pathological conditions?

In addition to sex-based differences in methylation-based age measurement, there has also been observed differential methylation in different ethnic groups, with most of the relevant work comparing African American and Caucasian individuals. Does it also follow that all ethnic groups have a group-specific set of CpG sites that are methylated differentially during aging? Conversely, is there a set of ethnicity-independent CpG sites that reflect the status of biological aging for all humans?

The above questions generally assume that the differences in aging observed in different sex and ethnicity groups is biological. This is not the only possibility; it is possible that there are inherent features of the clocks here examined that lead to those differential measurements. The above observed limited overlap in CpG sites may contribute to this inherent difference, and additionally the training data employed (many of the clocks reviewed here rely on regression analysis of a body of training data) may contribute to a group-related bias. Testing different clocks on the training data employed by other clocks may help to reveal any inherent differences in these clocks.

### 4.3 Biological age variables

It has been speculated that diet and lifestyle, among other modifiable features, contribute to differing rates of biological aging. Is diet a primary contributor to biological aging, or rather, is it an indicator of other socioeconomic factors that directly contribute to differences in biological aging? Similarly, physical activity has been found to modulate biological aging, but the underlying relationship between physical activity and biological age remains to be characterized. Some of the papers reviewed here explore biological aging changes in relation to pregnancy and gestation, while other work has considered the contribution of early life events to later biological aging status. What contributions do maternal health make to later biological aging? How do early life events influence biological aging: do they alter biological aging rates at the time of the event, or is it a delayed effect? In either case, is there an acute feature that can assess the biological aging contributions of a given early life event? While telomere length has been explored in a number of model organisms including non-vertebrate organisms, methylation-based clock development in model organisms has lagged. Is methylation status a human-specific, primate-specific, mammalian-specific, or general animal feature of biological aging? The answer to this expansive question will greatly influence what animal models are most relevant to the study of aging in human subjects.

## 5 Conclusion

We examined twelve and a half years of research on the role methylation plays in the process and measurement of biological aging. While methylation-based measurements are a widely used metric for assessing biological age changes, there are a number of outstanding questions into the relationships between methylation changes and the underlying changes or resulting consequences of aging. In particular, it remains to be determined whether a ‘universal clock’ is possible with blood-based methylation data, or even if blood-based measurements are the most revealing. There remains a great breadth of research opportunity in this field, with many intriguing possibilities for the questions and hypotheses outlined above. There remains room for great progress in both early detection and therapeutics based on methylation-derived biological age measurement.
